# Association between radical versus conservative surgery and short-term outcomes of hepatic cystic echinococcosis in Nyingchi, China: a retrospective cohort study

**DOI:** 10.1186/s12893-023-02000-y

**Published:** 2023-05-12

**Authors:** Liangping Huang, Benrong Zheng, Aduo, Ouzhulamu, Xi Li, Jianchun Yao

**Affiliations:** 1 Department of Drug and Medical Device Clinical Trial Office, The Second Affiliated Hospital of Shantou University Medical College, Shantou, China; 2 Department of Hematology and Oncology, Shenzhen Children’s Hospital of China Medical University, Shenzhen, China; 3 Physical Examination Center, Nyingchi People’s Hospital, Nyingchi, China; 4 Department of VIP Health Care Center, The Third Affiliated Hospital of Sun Yat-Sen University, Guangzhou, China; 5 Department of General Surgery, Nyingchi People’s Hospital, Nyingchi, China; 6 Department of Thyroid and Breast Surgery, The Third Affiliated Hospital of Sun Yat-Sen University, No.600 Tianhe Road, Guangzhou, 510630 China; 7 Department of Anesthesiology, Nyingchi People’s Hospital, Nyingchi, China; 8 Department of Anesthesiology, The First Affiliated Hospital of Shantou University Medical College, No. 57 Changping Road, Jinping District, Shantou, 515041 Guangdong Province China

**Keywords:** Hepatic cystic echinococcosis, Surgical procedure, Liver resection, Echinococcus granulosus, Tibet

## Abstract

**Background:**

Radical or conservative surgical treatment for hepatic Cystic Echinococcosis (hepatic CE) is controversial. We aimed to measure the association between radical surgery (RS) versus conservative surgery (CS) and short-term outcomes in our cohort.

**Methods:**

Medical records of hepatic CE patients’ demographic, clinical, radiological, operative and postoperative details who underwent surgical treatment between January 3, 2017 and January 3, 2018 at the Department of General Surgery, Nyingchi People's Hospital, Nyingchi, China, were retrieved and analyzed. The primary outcome was overall morbidity. The secondary outcomes included: (i) bile leakage; (ii) complications of lung, pleura, heart, liver, pancreas and biliary tract; (iii) incision infection and residual cavity abscess formation; (iv) anaphylactic reaction and shock; (v) tear of surrounding tissues; (vi) hospital and post-operative length of stay (LOS); (vii) length of surgery; (viii) blood loss during surgery. Multivariable logistic/linear regression models with various adjustment strategies for confounders were performed to evaluate the association.

**Results:**

A total of 128 hepatic CE patients were included with 82 (64.1%) and 46 (35.9%) receiving CS and RS, respectively. After fully adjusted, RS was associated with 60% lower risk of overall complication (aOR 0.4; 95%CI, 0.2–0.9) and 0.6-h shorter surgical time (aβ 0.4; 95%CI,-0.0–0.8) comparing to CS. However, RS was associated with more blood loss during surgery (aβ 179.3; 95%CI, 54.2–304.5).

**Conclusion:**

To conclude, RS was associated with a 60% reduction in developing overall complication in the short term, but may result in more blood loss during surgery than CS.

## Introduction

Hepatic echinococcosis is a global public health issue that has a significant negative impact on both health and economy [1, 2]. It is a zoonotic disease and liver is most commonly affected, the majority of which is Cystic Echinococcosis (CE) caused by Echinococcosis Granulosus infection, followed by Alveolar Echinococcosis (AE) caused by Echinococcosis Multilocularis [1, 3, 4]. It has a global endemic distribution pattern, with the Qinghai-Tibet plateau in China, South America, and Eastern Europe being particularly endemic regions [1, 2, 5]. Managements for hepatic CE are: (i) percutaneous treatment, (ii) surgery, (iii) antiparasitic drug, or (iv) watch and wait [6]. No optimal treatment is recommended for hepatic CE [6, 7]. Surgery, in conjunction with adjuvant anti-parasitic agent, is currently the primary method of CE treatment, particularly in cases of large, superficial, and/or complicated cysts [6–8]. In general, surgical managements are divided into radical surgery (RS), which includes total pericystectomy as well as liver resection, and conservative surgery (CS), which includes sub-total cystectomy and partial cystectomy [2, 3, 9]. The decision between RS and CS is debatable, because more invasive procedures are linked to increased rates of complications, but reduced rates of recurrence [1]. Furthermore, intensive therapy for a benign condition is frequently criticized [10]. Sizable numbers of studies have compared CS and RS treatment on hepatic CE and found RS favored the decrease of some short-term and/or long-term treatment outcomes [9, 11, 12]. However, the power of the evidence is insufficient because only hypothesis tests without adjusting for baseline characteristics can falsely reject the null hypothesis and lead to an incorrect conclusion [13]. On the other hand, controversial finding was observed in a retrospective study by El Malki HO et al. [14], which no statistically significant difference in outcomes was found between RS and CS after propensity score matching and multivariable logistic analysis.

The average prevalence rate of Echinococcosis in Qinghai-Tibet plateau of China approaches 10% (with a range of 0.8 to 11.9%) [15]. Communities of herdsmen, owned and stray dogs, and low levels of awareness of echinococcosis are risk factors for the prevalence [16]. Starting from 2005, the Chinese Government has implemented a hydatid disease control program in endemic area. In the last 5 years, Nyingchi city, which is situated in the southeast of Qinghai-Tibet plateau, has made great effort to manage and control Echinococcosis endemic. This encourages local investigation and sparks interest in Echinococcosis clinical research.

Therefore, in order to strengthen the power of the evidence, add evidence to the existing literatures, and especially for self-improvement, we retrospectively collected hospital records of hepatic CE to analyze the association between CS versus RS and short-term outcomes using a multivariate regression model with adjustment for potential confounders.

## Methods

Between January 3, 2017, and January 3, 2018, data from consecutive Echinococcosis patients who underwent surgical treatment at the Department of General Surgery, Nyingchi People's Hospital, Nyingchi, Tibet, China, were reviewed retrospectively. Patients with a diagnosis of hepatic CE who were admitted for surgical treatment met the inclusion criteria. The following patients met the exclusion criteria: (i) patients without hepatic cysts; (ii) patients with hepatic Alveolar Echinococcosis (hepatic AE) diagnoses or without information to distinguish hepatic CE from hepatic AE; and (iii) patients whose surgical records and study results were lacking. This study has been approved by the Ethics Committee of Nyingchi People’s Hospital, Nyingchi, Tibet, China (Ethic No. 2022-8th). The requirement for inform consent was waived because the data were processed, anonymized, and collected retroactively.

The medical records of demographic, clinical, radiological, operative and postoperative details were retrieved for analysis. Clinical history, physical examination, abdominal ultrasonography, and/or radiographic studies were used in all patients to establish the diagnosis of hepatic CE. Anatomical details of cysts and its surrounding structures were identified by Computed tomography (CT). Serological tests weren't frequently employed.

The primary outcome of our study was the risk of overall morbidity, which is defined as newly emerging diagnosis or abnormal findings from auxiliary examinations with clinical significance from the start of the surgery to the moment of discharge [9]. The secondary outcomes were operation-related complications: (i) Bile leakage, defined as biliary drainage through the abdominal drains [17]; (ii) Complications of lung, pleura, heart, liver, pancreas and biliary tract, which were defined by relevant symptoms, lab findings and imaging findings; (iii) Infection of the incision and residual cavity abscess formation; (iv) Anaphylactic reaction and shock; (v) Tear of surrounding tissues during surgery; (vi) Hospital and post-operative length of stay (LOS); (vii) Length of surgery; (viii) Blood loss and blood loss ≥ 500 mL during surgery. The exposure variables were CS or RS [2, 3, 9]. The selection between CS or RS was left to the surgeons based on the judgement of preoperative and intraoperative findings as well as the surgeon’s expertise. Each patient received thorough counseling and information before operation consent was completed. All patients in our study underwent open surgery via right subcostal and upper midline incision for hydatid cyst of the right and left lobe, respectively. The brief surgical procedure was as follows: surrounding area protection, cyst content evacuation and decompression, endo-cystectomy, bile leak management, pericystectomy or lobectomy, and external drainage. Whenever necessary, the Pringle maneuver was applied. The open cyst resection technique [6, 10] was adopted in all RS cases. Albendazole tablet, 10-15 mg/kg per day was prescribed according to the 2015 edition of Expert Consensus on Diagnosis and Treatment of Hepatic Echinococcosis in China, which is in accordance with the WHO/IWGE guideline [8]. Before surgery, albendazole was given for all patients for 3–7 days. After surgery, albendazole was not continuous after RS. For CS, albendazole was not given for inactive cysts (CE4, CE5), but was continuous for the active or transitional cysts (CL, CE1-3) for 3 to 12 months. The covariates included age, sex, previous surgical history of Hepatic Hydatid Echinococcosis, number of cysts, cyst diameter, cyst location, infected cyst, and Pringle maneuver. All covariate information was obtained at baseline. The rationale for covariates inclusion was mainly based on previous work, our clinical experience, and prior literature that also used post-operative outcome as the outcome variable [3, 9].

### Statistical analysis

Baseline characteristics were presented as the mean ± standard deviation (SD) for continuous variables and as frequency (%) for categorical variables. The differences in demographics and clinical characteristics were compared using t-test or chi-square test, as appropriate. The association between CS/RS and all covariates with overall morbidity was evaluated by univariable analysis. Variables with a *p* value ≤ 0.2 in the univariate analysis would be included in the multivariable regression model [18]. The ORs (outcome as dichotomous variable) or βs (outcome as continuous variable) of the primary and secondary outcomes were estimated by the CS and RS as exposure variables using multivariable logistic regression models (outcome as dichotomous variable) or multivariable linear regression models (outcome as continuous variable) without adjustment, and with adjustment for age and sex (Minimally Adjusted), and with adjustment for age, sex, previous surgical history of hepatic hydatid disease, cyst diameter, and infected cyst (Fully Adjusted). A two-tailed *P* value < 0.05 was considered statistically significant. All analyses were performed using Empower Stats statistical software (http://www.empowerstats.com, X&Y Solutions, Inc., Boston, MA, USA) and the statistical package R (version 4.2.0, http://www.r-project.org).

## Results

**Fig. 1:**
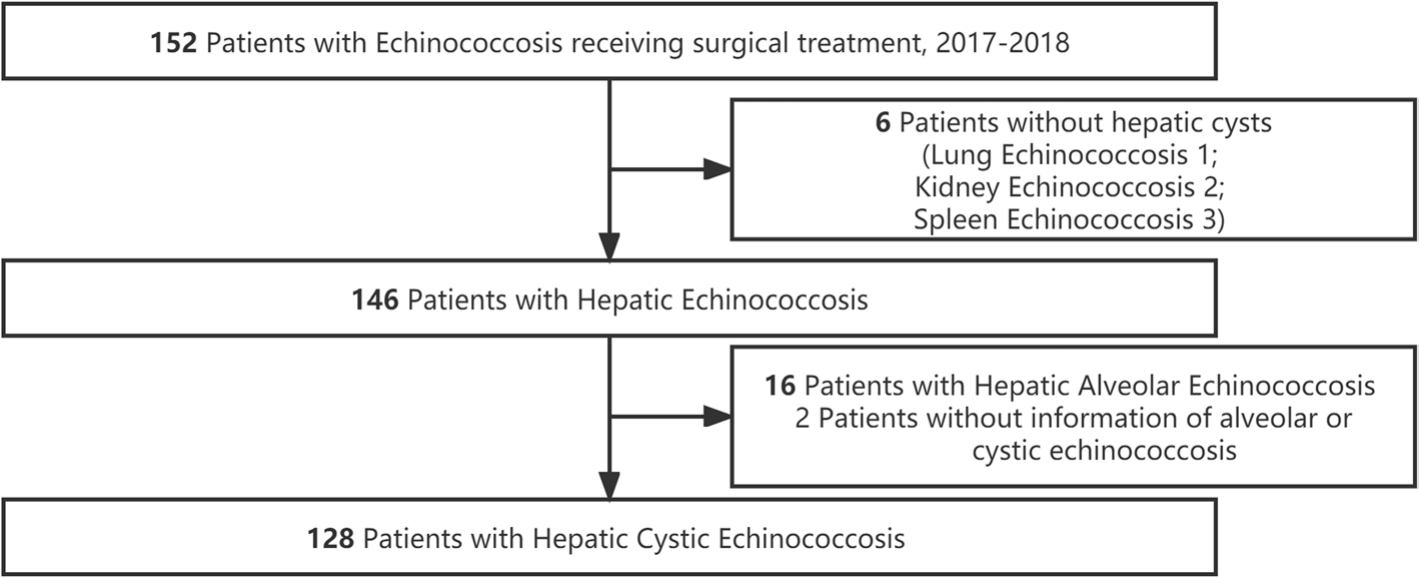
Flow chart of recruitment process

Out of 152 patients with Echinococcosis, 24 individuals were excluded (6 without lesion in the liver, 16 diagnosed as hepatic AE, 2 without information to distinguish hepatic CE from hepatic AE), leaving 128 hepatic CE individuals eligible for subsequent analysis (Fig. 1). The demographic and clinical characteristics were described in Table 1. Based on the surgical procedure, the cohort was divided into CS group and RS group. Eighty-two patients underwent CS and 46 underwent RS. Baseline characteristics were comparable between the two groups. The mean age of the cohort was 41.3 ± 14.1 years with female preponderance (*n* = 68, 53.1%). Abdominal signs and symptoms were most commonly presented. The severity of abdominal pain ranged from mild right upper quadrant discomfort, abdominal distention, to dull pain (data not shown). The average diameter of cysts was 10 ± 3.6 cm. Multiple cysts and infected cysts were presented in 22 (17.2%) and 15 (11.7%) of patients, respectively. Cysts affecting bilateral lobes accounted for 11.4%, with the remainder affected unilateral lobe (right posterior lobe 45.5%, right anterior lobe 22.8%, left lobe 20.8%; 5 individuals with right lobe cysts had missing data on either the right anterior or right posterior). Twenty (15.7%) had previous surgical history for hepatic Echinococcosis.

**Table 1:** Demographics and clinical characteristics of the patients

	Total (*n* = 128)	CS (*n* = 82)	RS (*n* = 46)	*P*
Age, mean (SD), y	41.3 (14.1)	42.8 (13.8)	38.7 (14.3)	0.111
Sex, No. (%)				0.872
Male	60 (46.9%)	38 (46.3%)	22 (47.8%)	
Female	68 (53.1%)	44 (53.7%)	24 (52.2%)	
Abdominal symptoms ^a^, No. (%)				0.442
No	21 (16.4%)	15 (18.3%)	6 (13.0%)	
Yes	107 (83.6%)	67 (81.7%)	40 (87.0%)	
Abdominal signs ^b^, No. (%)				0.963
No	22 (17.2%)	14 (17.1%)	8 (17.4%)	
Yes	106 (82.8%)	68 (82.9%)	38 (82.6%)	
Fever, No. (%)				0.180
No	127 (99.2%)	82 (100.0%)	45 (97.8%)	
Yes	1 (0.8%)	0 (0.0%)	1 (2.2%)	
Total bilirubin, categorial ^c^, No. (%)				0.454
< 17.1 umol/L	100 (81.3%)	62 (80.5%)	38 (82.6%)	
> = 17.1, < 34 umol/L	20 (16.3%)	14 (18.2%)	6 (13.0%)	
> = 34 umol/L	3 (2.4%)	1 (1.3%)	2 (4.3%)	
Previous surgical history of Hepatic Echinococcosis, No. (%)				0.373
No	107(84.3%)	70 (86.4%)	37 (80.4%)	
Yes	20(15.7%)	11 (13.6%)	9 (19.6%)	
WHO/IWGE classification of cysts, No. (%)				0.104
CL	3 (3.6%)	1 (1.9%)	2 (6.7%)	
CE1	11 (13.1%)	10 (18.5%)	1 (3.3%)	
CE2	20 (23.8%)	14 (25.9%)	6 (20.0%)	
CE3	24 (28.6%)	17 (31.5%)	7 (23.3%)	
CE4	24 (28.6%)	11 (20.4%)	13 (43.3%)	
CE5	2 (2.4%)	1 (1.9%)	1 (3.3%)	
Concomitant extra-hepatic cysts, No. (%)				-
Lung	1 (0.8%)	1 (1.2%)	-	
Kidney	1 (0.8%)	-	1 (2.2%)	
Spleen	1 (0.8%)	-	1 (2.2%)	
Peritoneal cavity	4 (3.1%)	2 (2.4%)	2 (4.3%)	
Cyst location, No. (%)				0.218
Left lobe	25 (20.3%)	13 (16.7%)	12 (26.7%)	
Right anterior lobe	28 (22.8%)	16 (20.5%)	12 (26.7%)	
Right posterior lobe	56 (45.5%)	41 (52.6%)	15 (33.3%)	
Bilateral lobes	14 (11.4%)	8 (10.3%)	6 (13.3%)	
Cyst diameter, mean (SD), cm	10.1 (3.6)	10.5 (3.6)	9.5 (3.4)	0.148
Cyst diameter, WHO categorical, No. (%)				0.793
< 5 cm	2 (1.6%)	1 (1.2%)	1 (2.2%)	
> = 5, < 10 cm	52 (40.6%)	32 (39.0%)	20 (43.5%)	
> = 10 cm	74 (57.8%)	49 (59.8%)	25 (54.3%)	
No. of cyst, No. (%)				0.593
1	106 (82.8%)	69 (84.1%)	37 (80.4%)	
Multiple (2 and more)	22 (17.2%)	13 (15.9%)	9 (19.6%)	
Infected cysts, No. (%)				0.171
No	113 (88.3%)	70 (85.4%)	43 (93.5%)	
Yes	15 (11.7%)	12 (14.6%)	3 (6.5%)	

Abbreviations: *CS* Conservative surgery, *RS* Radical surgery, *SD* Standard deviation, *WHO-IWGE* WHO-informal working group on echinococcosis

^a^ Abdominal symptoms: including abdominal discomfort, abdominal pain, nausea, vomiting

^b^ Abdominal signs: including abdominal tenderness, abdominal lump, hepatomegaly

^c^ Due to poor medical records, blood total bilirubin levels are used to represent jaundice, which < 17.1 umol/L, 17.1 ~ 34 umol/L and >  = 34 umol/L indicates normal, occult jaundice and gross jaundice, respectively

The operative-related complications were listed in Table 2. Fifty-five (43.0%) patients developed complications, with bile leakage, pleural and lung complications, and post-operative hypoalbuminemia being the most common. Overall morbidity was predominant in the CS group. In the univariable analysis (Table 3), RS was negatively associated with overall morbidity, while infected cysts were positively associated with overall morbidity.

**Table 2:** Operation-related complications of the patients

	Total (*n* = 128)	CS (*n* = 82)	RS (*n* = 46)	*P*
Overall morbidity, No. (%)				0.012
No	73 (57.0%)	40 (48.8%)	33 (71.7%)	
Yes	55 (43.0%)	42 (51.2%)	13 (28.3%)	
Bile leakage, No. (%)				0.075
No	97 (75.8%)	58 (70.7%)	39 (84.8%)	
Yes	31 (24.2%)	24 (29.3%)	7 (15.2%)	
Pleural complication, No. (%)				0.682
No	115 (89.8%)	73 (89.0%)	42 (91.3%)	
Yes	13 (10.2%)	9 (11.0%)	4 (8.7%)	
Lung complication, No. (%)				0.684
No	118 (92.2%)	75 (91.5%)	43 (93.5%)	
Yes	10 (7.8%)	7 (8.5%)	3 (6.5%)	
Hepatic failure, No. (%)				0.676
No	126 (98.4%)	81 (98.8%)	45 (97.8%)	
Yes	2 (1.6%)	1 (1.2%)	1 (2.2%)	
Heart complication, No. (%)				0.180
No	127 (99.2%)	82 (100.0%)	45 (97.8%)	
Yes	1 (0.8%)	0 (0.0%)	1 (2.2%)	
Acute cholangitis, No. (%)				0.452
No	127 (99.2%)	81 (98.8%)	46 (100.0%)	
Yes	1 (0.8%)	1 (1.2%)	0 (0.0%)	
Abscess of residual cavity, No. (%)				0.452
No	127 (99.2%)	81 (98.8%)	46 (100.0%)	
Yes	1 (0.8%)	1 (1.2%)	0 (0.0%)	
Incision infection, No. (%)				0.676
No	126 (98.4%)	81 (98.8%)	45 (97.8%)	
Yes	2 (1.6%)	1 (1.2%)	1 (2.2%)	
Shock, No. (%)				0.924
No	125 (97.7%)	80 (97.6%)	45 (97.8%)	
Yes	3 (2.3%)	2 (2.4%)	1 (2.2%)	
Anaphylactic reaction, No. (%)				0.452
No	127 (99.2%)	81 (98.8%)	46 (100.0%)	
Yes	1 (0.8%)	1 (1.2%)	0 (0.0%)	
Acute pancreatitis, No. (%)				0.924
No	125 (97.7%)	80 (97.6%)	45 (97.8%)	
Yes	3 (2.3%)	2 (2.4%)	1 (2.2%)	
Tear of common bile duct, No. (%)				0.452
No	127 (99.2%)	81 (98.8%)	46 (100.0%)	
Yes	1 (0.8%)	1 (1.2%)	0 (0.0%)	
Tear of diaphragm, No. (%)				0.452
No	127 (99.2%)	81 (98.8%)	46 (100.0%)	
Yes	1 (0.8%)	1 (1.2%)	0 (0.0%)	
Tear of colon, No. (%)				0.452
No	127 (99.2%)	81 (98.8%)	46 (100.0%)	
Yes	1 (0.8%)	1 (1.2%)	0 (0.0%)	
Hypoalbuminemia, No. (%)				0.683
No	115 (89.8%)	74 (90.2%)	41 (89.1%)	
Yes	13 (10.2%)	8 (9.8%)	5 (10.9%)	

Abbreviations: *CS* Conservative surgery, *RS* Radical surgery

**Table 3:** Univariable analysis of factors associated with overall morbidity

	Statistics	Combined complication	*p*-value
	No. (%)	OR (95%CI)	
Age categorical, y			
< = 20	11 (8.6%)	Ref	
> 20, < = 60	109 (85.2%)	1.1 (0.2—5.2)	0.951
> 60	8 (6.2%)	1.8 (0.1—23.3)	0.660
Sex			
Male	60 (46.9%)	Ref	
Female	68 (53.1%)	0.7 (0.3—1.4)	0.319
No. of cysts			
1	106 (82.8%)	Ref	
2 and more	22 (17.2%)	0.6 (0.2—1.7)	0.371
Previous surgical history of hepatic Echinococcosis			
No	107 (84.3%)	Ref	
Yes	20 (15.7%)	0.4 (0.1—1.1)	0.064
Cyst diameter, categorial			
< 10 cm	54 (42.2%)	Ref	
10 cm and more	74 (57.8%)	1.3 (0.6—2.7)	0.492
Surgical approach			
CS	82 (64.1%)	Ref	
RS	46 (35.9%)	0.4 (0.2—0.8)	0.017
Cyst location			
Left lobe	25 (20.3%)	Ref	
Right anterior lobe	28 (22.8%)	1.1 (0.4—3.4)	0.877
Right posterior lobe	56 (45.5%)	2.0 (0.7—5.3)	0.178
Bilateral lobes	14 (11.4%)	0.7 (0.2—2.9)	0.597
Infected cyst			
No	113 (88.3%)	Ref	
Yes	15 (11.7%)	3.2 (1.0—10.0)	0.051
Pringle maneuver			
No	111 (86.7%)	Ref	
Yes	17 (13.3%)	0.7 (0.3—2.1)	0.572

Abbreviations: *OR* Odds ratio, *95%CI* 95% confidential interval, *Ref.* Reference, *CS* Conservative surgery, *RS* Radical surgery

We analyzed the association between CS vs RS and short-term outcomes using various covariates adjustment strategies in the multivariable logistic or linear regression model (Table 4). RS was associated with a lower risk of overall morbidity (OR 0.4; 95%CI, 0.2—0.8; *p* = 0.013) in the non-adjusted model. The result was robust after minimally adjusting for age and sex (adjusted OR 0.4; 95%CI, 0.2—0.8; *p* = 0.017). After fully adjusting for age, sex, previous surgical history of hepatic hydatid disease, cyst diameter, and infected cyst, the result remained resilient (adjusted OR 0.4; 95%CI, 0.2—0.9; *p* = 0.029). On the other hand, RS was associated with higher level of blood loos. Specifically, in the non-adjust model, RS was associated with more than 160 ml higher levels of blood loss during surgery (β 167.0; 95%CI, 37.4—296.5; *p* = 0.013); after fully adjusted, the result remained robust (adjusted β 179.3; 95%CI, 54.2—304.5; *p* = 0.006). Additionally, RS was associated with shorter length of surgery (β 0.5; 95%CI, 0.0—0.9; *p* = 0.039); however, after adjustment, the effect was somewhat variable (fully adjusted β 0.4; 95%CI,-0.0—-0.8; *p* = 0.070), suggesting other factors may have an impact on the length of surgery. Notably, a trend towards lower risk of bile leakage (fully adjusted OR 0.5; 95%CI, 0.2—1.2; *p* = 0.125), but higher risk of blood loss ≥ 500 mL (fully adjusted OR 3.0; 95%CI, 0.9—9.9; *p* = 0.074) during surgery was observed. In our cohort, no differences were found in terms of lung complication, pleural complication, and hospital or post-surgical LOS between the two groups.

**Table 4:** Multivariable analysis of the association between RS and short-term outcomes with reference to CS

	Non-adjusted ^b^		Minimally Adjusted ^b^		Fully Adjusted ^b^	*p*-value
	OR/β (95%CI)	*p*-value	aOR/aβ (95%CI)	*p*-value	aOR/aβ (95%CI)	
Overall morbidity	0.4 (0.2, 0.8)	0.013	0.4 (0.2, 0.8)	0.017	0.4 (0.2, 0.9)	0.029
Bile leakage	0.4 (0.2, 1.1)	0.080	0.5 (0.2, 1.2)	0.104	0.5 (0.2, 1.2)	0.125
Lung complication	0.7 (0.2, 3.0)	0.684	0.7 (0.2, 2.8)	0.586	0.7 (0.2, 2.9)	0.602
Pleural complication	0.8 (0.2, 2.7)	0.683	0.8 (0.2, 2.7)	0.681	0.8 (0.2, 3.0)	0.783
Intra-operative blood loss ^a^	167.0 (37.4, 296.5)	0.013	175.3 (44.5, 306.1)	0.010	179.3 (54.2, 304.5)	0.006
Intra-operative blood loss ≥ 500 mL	2.8 (0.9, 8.7)	0.083	2.9 (0.9, 9.2)	0.075	3.0 (0.9, 9.9)	0.074
Length of surgery ^a^	0.5 (0.0, 0.9)	0.039	0.4 (-0.0, 0.8)	0.066	0.4 (-0.0, 0.8)	0.070
Hospital LOS ^a^	0.1 (-1.8, 2.1)	0.884	0.2 (-1.8, 2.2)	0.865	0.8 (-1.1, 2.7)	0.399
Post-surgical LOS ^a^	0.6 (-1.2, 2.5)	0.500	0.7 (-1.1, 2.6)	0.440	1.1 (-0.7, 2.9)	0.230

Abbreviations: *RS* Radical surgery, *CS* Conservative surgery, *OR* Odds ratio, *aOR* Adjusted OR, *aβ* Adjusted β, *95%CI* 95% confidential interval, *LOS* Length of stay

^a^ Continuous variates

^b^ Non-adjusted: we adjusted for none; Minimally adjusted: we adjusted for age, sex; Fully adjusted: we adjusted for age, sex, previous surgical history of hepatic Echinococcosis, cyst diameter, and infected cyst

## Discussion

This retrospective cohort study investigated the association between RS versus CS and short-term outcomes. In comparison to CS, RS was associated with 60% lower risk of developing overall complication and 0.6-h shorter surgical time, as well as a trend towards protective effect on bile leakage. However, RS was associated with nearly 180 ml higher level of blood loss during surgery, and a trend of twofold increased risk of developing 500 mL or more blood loss.

In terms of overall morbidity, result in our study is consistent with Georgiou GK, et al. [9] and Farhat W et al. [19]. Georgios K, et al. [9], conducted a retrospective cohort study of 232 patients with hepatic cystic echinococcosis and found a lower rate of morbidity and post-operative complication in the RS group. Farhat W et al. [19], by using paired comparison analysis in a cohort of 914 patients, observed lower rate of overall morbidity in the RS group before and after paired-match. The same outcome was observed in our cohort. However, we further calculate the exact effect size of a 60% reduction in overall morbidity in RS group comparing to CS group. Our result was comparable to those of a recent meta-analysis by Pang Q et al. [20], who recruited 19 studies with 1853 and 2274 patients receiving RS and CS, respectively. In contrast, El Malki HO et al. [14] observed no association between RS versus CS and overall postoperative complications after propensity score matching and multivariate logistic analysis, which contradicts the finding in our cohort. We speculate that the main reasons for the different results are: firstly, the regional and periodical disparities of study populations: patients in our cohort were all inhabitants in Qinghai-Tibet plateau between January 3, 2017 and January 3, 2018, whereas patients in El Malki HO et al.’s cohort were between January 1990 and December 2010 without details of nationality; secondly, the inconsistency in algorithms: multi-regression models were used to control confounders in our study, whereas propensity score matching analysis were used in El Malki HO et al.’s study.

Post-surgical bile leakage is risk factor of morbidity after surgery [3, 21, 22]. The incidence is reported to be ranged from 8.5% to 64.5% in CS group in different literatures [3, 23–25]. Georgiou GK, et al. [9] found a lower rate of developing post-operative bile leak in the RS group than CS group (3.48% vs 8%). Pang Q et al. [20] found that RS was associated with lower risk of bile leakage (OR 0.24; 95%CI,0.14–0.39). In our study, a fully adjusted odds ratio of 0.5 indicated a protective effect tendency in the RS group; however, the 95%CI 0.2 to 1.2 was “wide”. As the 95%CI may be constrained by sample size, we would rather not to deny the result. In the future, we will expand the sample size for further statistics.

In terms of length of surgery, controversial findings were presented. Pang Q et al. [20] found no difference in length of surgery between CS and RS group. Efanov M et al. [11], Farhat W et al. [19] observed that RS was more time consuming. Also, Akbulut S et al. [25] estimated about 30 min shorter operation time in the CS group. However, out of our expectation, we found 0.6 h shorter operation time in the RS group. As for blood loos during surgery, result in our study is in accordance with Farhat W et al. [19] who observed a higher incidence of intraoperative bleeding in the RS group. On the contrary, Daradkeh S et al. [26], found no significant difference in intraoperative blood loss in a prospective randomized control study involving 32 patients. These conflicting findings regarding length of surgery and blood loss may be attributed to various sites, study population, surgeons and surgical principles [27]. We followed the principle to remove the peri-cyst as much as possible on the basis of retaining normal liver tissue as much as possible, which resulted in a fact that CS were performed in more complicated cases, and thus, more time consuming and more effort to avoid blood loss; while RS were performed more aggressively, and thus, more blood loss. Furthermore, volume of intraoperative blood loss was an independent poor predictor for not only short-term [28], but also long-term [29] prognosis of hepatectomy; therefore, we cannot determine RS was truly superior to CS in our study without evaluating the long-term outcomes.

The advantages of our study are: (i) Compared with previous cross-sectional and case–control studies, this study was designed as a cohort study; (ii) Compared with previous studies with only groups comparison using t-test, chi-square test or Mann–Whitney U test, this study performed multivariate regression analysis that allowed adjustment for different confounding factors and obtaining accurate effect sizes.

The limitations of our study are: (i) Patients with hepatic AE were excluded from the study. Therefore, findings in this study cannot be applied to hepatic AE patients. (ii) This study only examines the short-term outcomes, which cannot be used to address long-term benefit of RS. (iii) This is a retrospective observational study, so confounding is inevitable. Even though we rigorously adjusted for confounding, we were only able to adjust measurable confounders, but not unmeasurable ones. (iv) Limited by the nature of observational studies, we could only observe associations, but not verify causality. Therefore, future prospective cohort studies or well-designed randomized control studies are warranted to validate our findings.

## Conclusion

To conclude, RS was associated with a 60% reduction in developing short-term overall complication, but may result in more blood loss during surgery than CS.

## Data Availability

The data that support the findings of this study are available on request from the corresponding author.

## Abbreviations

CE: Cystic echinococcosis
AE: Alveolar echinococcosis
RS: Radical surgery
CS: Conservative surgery
LOS: Length of stay
CT: Computed tomography
SD: Standard deviation
WHO-IWGE: WHO-informal working group on echinococcosis
OR: Odds ratio
95%CI: 95% Confidential interval
Ref.: Reference
aOR: Adjusted OR
aβ: Adjusted β
